# Case Report: Delayed Ventricular Pseudoaneurysm After Radiofrequency Ablation of Left Posteromedial Papillary Muscle Ventricular Tachycardia

**DOI:** 10.3389/fcvm.2022.887190

**Published:** 2022-06-15

**Authors:** Min Kim, Yoon Jung Park, Hee Tae Yu, Tae-Hoon Kim, Jae-Sun Uhm, Boyoung Joung, Hui-Nam Pak, Moon-Hyoung Lee

**Affiliations:** ^1^Division of Cardiology, Chungbuk National University Hospital, Cheongju, South Korea; ^2^Division of Cardiology, Yonsei University Health System, Seoul, South Korea

**Keywords:** ventricular tachycardia, catheter ablation, pseudoaneurysm, complication, intracardiac echocardiography

## Abstract

A 74-year-old woman presented with incessant wide complex tachycardia that was refractory to cardioversions. Successful radiofrequency catheter ablation was performed on the left ventricular posteromedial papillary muscle. An inaudible steam pop has occurred during the procedure, but we confirmed that there were no complications during the procedure and short-term follow-up of echocardiography. Two months after the procedure, an asymptomatic pseudoaneurysm was identified at the ablation site that had not been observed in the short-term follow-up.

## Introduction

Radiofrequency ablation is a surgical procedure used to resolve ventricular arrhythmias originating from the papillary muscle of the left ventricle (LV). Although usually challenging due to anatomical constraints and catheter instability, intracardiac echocardiography assists in achieving a high procedural success rate and immediate identification of procedural complications. The risk of cardiac perforation in catheter ablation of ventricular arrhythmia is less than 1%, which is relatively small compared to the risk of atrial catheter ablation and inaudible steam pop during catheter ablation is the most plausible mechanism for this complication (1–3). Delayed pseudoaneurysm in the LV is a very rare manifestation of catheter ablation (4), and information about its prognosis and management is limited, especially when surgical treatment is not necessary. Here we report a case of ventricular tachycardia (VT) inducing a delayed LV pseudoaneurysm on the radiofrequency catheter ablation site of the left posteromedial papillary muscle, which was absent during the short-term echocardiographic follow-up.

## Case Presentation

**FIGURE 1:**
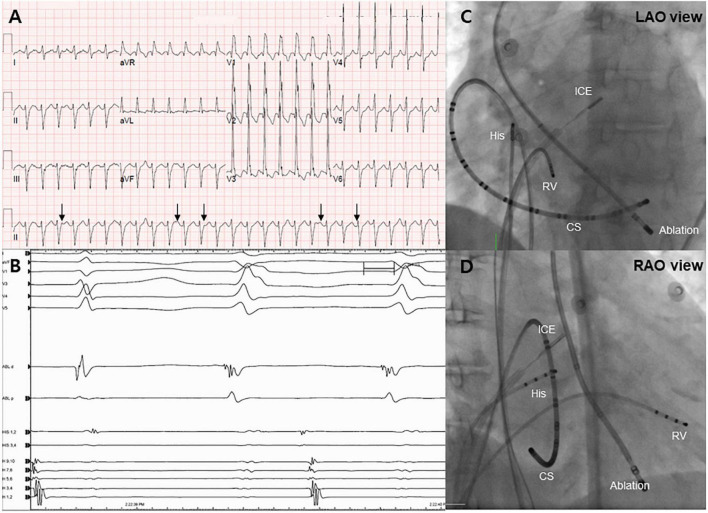
Electrocardiogram and endocardial mapping. **(A)** Electrocardiogram of monomorphic ventricular tachycardia showing ventriculoatrial dissociation (black arrows). **(B)** Intracardiac electrogram revealing an early signal (−40 ms) before the QRS complex during the ventricular tachycardia rhythm. **(C,D)** Right and left anterior oblique projections of endocardial mapping using intracardiac echocardiography. CS, coronary sinus; RV, right ventricle.

A 74-year-old woman presented to the emergency department with symptomatic wide complex tachycardia in the absence of hemodynamic deterioration. On admission, electrocardiography demonstrated monomorphic VT with a right bundle branch block pattern, a Q wave in lead V_1_, left superior axis deviation, and ventriculoatrial dissociation, suggesting an exit site in the posteromedial papillary muscle region of the left ventricle (Figure 1A). Despite the patient undergoing electrical and pharmacological cardioversions, the VT remained unresponsive. The patient had a history of hypertension with antihypertensive drug use but no history of structural heart disease or tobacco or alcohol use. On the baseline examination, transthoracic echocardiography estimated LV end-diastolic dimension and LV ejection fraction to be 62 mm and 26%, respectively. Cardiac computed tomography (CT) revealed no significant stenotic lesions in the coronary arteries and no structural heart disease (Supplementary Figures 1A,B), indicating tachycardia-induced cardiomyopathy.

**FIGURE 2:**
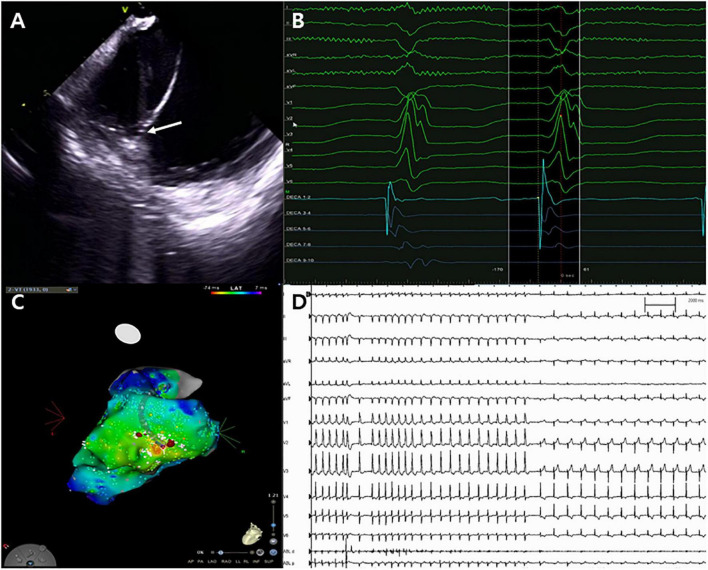
Catheter ablation for ventricular tachycardia. **(A)** Successful ablation site on the left posteromedial papillary muscle (white arrow). **(B,C)** Unipolar Q signal on successful ablation site and three-dimensional activation map. **(D)** Termination of ventricular tachycardia with steam pops.

**FIGURE 3:**
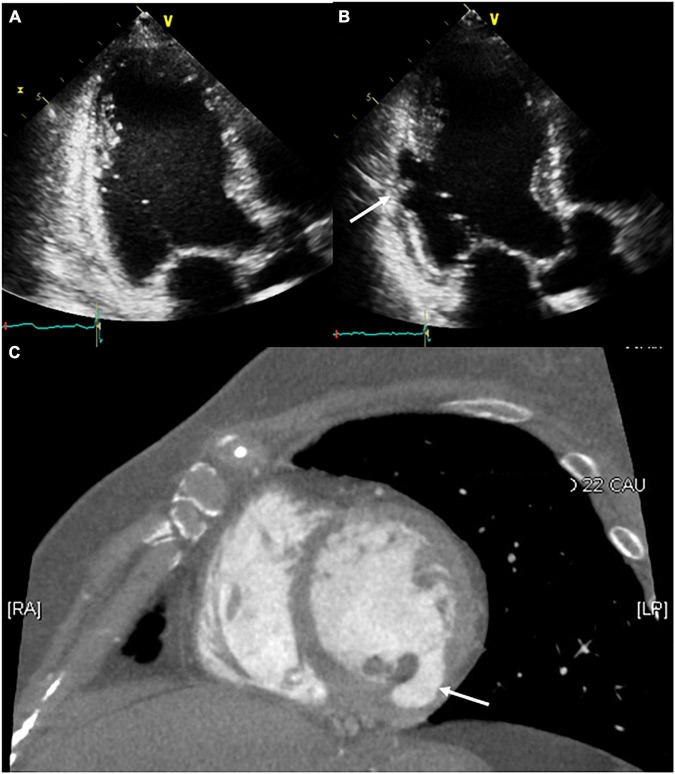
Serial follow-up of the ablation site using transthoracic echocardiography. **(A)** The day after the procedure, no structural changes around the ablation site were observed. **(B,C)** After 2 months of follow-up, the development of a pseudoaneurysm on the ablation site (white arrow) was confirmed through transthoracic echocardiography and contrast-enhanced computed tomography.

Following admission to the cardiac intensive care unit, the patient underwent a percutaneous left-sided stellate ganglion block with bupivacaine, a cardiac sympathetic intervention suppressing sympathetic activity for controlling VT. However, the cardiac sympathetic intervention was unsuccessful. Given that the VT remained unresolved and the driving cause of electrical instability was unclear, radiofrequency catheter ablation was decided as the most appropriate treatment strategy. Endocardial electroanatomical mapping of the LV using a CARTO-3 system (Biosense Webster) was performed through both transseptal and retroaortic approaches using a multi-electrode catheter. The procedure was performed using intracardiac echocardiography (ICE) through a transseptal approach and viewing the LV structure in real-time. The ablation catheter was positioned through the retroaortic approach. During endocardial mapping, intracardiac electrograms showed fractionated potentials around the posteromedial papillary muscle with the earliest activation (−40 ms) (Figures 1B–D). Endocardial bipolar voltage mapping revealed no scarring (0.5–1.5 mV), and the activation map was consistent with an exit site in the posteromedial papillary muscle region. Under direct ICE visualization (Figure 2A), endocardial ablation using an irrigated-tip catheter (SmartTouch, Biosense Webster) and 40 W was applied to target the earliest site that showed the initial Q wave in the unipolar signal with 10–15 g of contact force (Figures 2B,C). During the first ablation, the VT was terminated for 30 s with an inaudible steam pop recorded by ICE (Figure 2D and Supplementary Video 1), and a sudden rise in electrical impedance was not noted. After confirming that no evidence of perforation or tissue defects were present in the first ablation site, multiple circumferential ablations were performed in the area surrounding the posteromedial papillary muscle without further steam pops. Repeated programmed electrical stimulation failed to induce VT following the ablation. Before the end of the procedure, the ICE operator confirmed that there was no evidence of procedure-related complications. The transthoracic echocardiography performed the next day showed no evidence of mitral regurgitation or tissue defects in the ablation sites (Figure 3A).

After 2 months of follow-up, the patient remained free of VT. Echocardiography showed improved LV systolic function with LV ejection fraction of 50% and a decreased LV size with LV end-diastolic dimension of 52 mm, but a pseudoaneurysm (26 × 9 mm) that has the ratio of the maximum diameter of the orifice to the maximum internal diameter of the cavity was lesser than 0.50 was identified at the base of the LV posteromedial papillary muscle where a steam pop had occurred without pericardial effusion (Figure 3B and Supplementary Video 2). The patient was asymptomatic; therefore, she was referred to the outpatient clinic for observational follow-up during the following year without anticoagulation or antiplatelet agents; the patient underwent no further interventions. One year later, cardiac CT revealed that the pseudoaneurysm that typically has a neck narrower than the diameter of the aneurysm remained present but showed no signs of a size change or other complications such as thrombus formation (Figure 3C).

## Discussion

Left ventricle pseudoaneurysm is a very rare complication of catheter ablation for ventricular arrhythmias that occurs regardless of the presence or absence of structural heart disease (4, 5). It is a form of cardiac rupture concealed by the adherent pericardium or scar tissue and has a wide range of clinical manifestations (6). Identifying LV pseudoaneurysm is often difficult, but very important because it may cause significant complications including cardiac perforation (2), hemodynamic deterioration by compressing adjacent coronary arteries (7), and thromboembolism (8). To reduce structural defects during catheter ablation, careful monitoring of the ablation parameters, including tissue temperature changes, impedance changes, electrical potentials, and catheter position, is essential for all team members and the primary operator.

It is often challenging to differentiate between LV pseudo aneurysms and true aneurysms. Although magnetic resonance imaging is the most useful for differentiation, it is still helpful for differentiation by comparing the ratio of end-systolic orifice diameter to maximal aneurysmal diameter through echocardiography or CT. The presence of turbulent flow by pulsed Doppler at the neck of a cavity or within the cavity by echocardiography can help differentiation (9).

In the case reported here, although the presence of turbulent flow by pulsed Doppler was unclear, other echocardiography and CT findings suggested a pseudoaneurysm. Most plausible cause of the pseudoaneurysm was an inaudible steam pop that occurred during the ablation. A steam pop occurs by the myocardial explosion when the tissue temperature reaches 100°C high enough to cause tissue vaporization and gas production; by ICE visualization, it appears as a sudden hyperechogenic intramyocardial microbubble formation around the catheter. It is a potentially life-threatening complication of radiofrequency ablation because it can cause structural defects (10). Intraprocedural ICE and transthoracic echocardiogram 1 day after the procedure revealed no tissue defects around the ablation site and no pericardial effusion. However, echocardiography performed at 2 months of follow-up confirmed the delayed development of a pseudoaneurysm at the first ablation site. We speculated that an inaudible steam pop cause both acute denuding and delayed endocardial necrosis. Here we reported a rare case of delayed LV pseudoaneurysm confirmed by echocardiography and cardiac CT that developed on an inaudible steam pop site occurring during ablation for the treatment of VT originating from the papillary muscle of the LV. However, magnetic resonance imaging is thought to be more useful when considering the diagnosis and follow-up of structural defects such as pseudoaneurysm. Serial imaging-based follow-up can facilitate the identification of pseudoaneurysms with delayed development, even when intraprocedural and short-term imaging-based follow-up confirms no procedure-related complications.

## Data Availability Statement

The original contributions presented in this study are included in the article/Supplementary Material, further inquiries can be directed to the corresponding author.

## Ethics Statement

Written informed consent was obtained from the participant for the publication of this case report. Written informed consent was obtained from the participant for the publication of any potentially identifiable images or data included in this article.

## Author Contributions

MK and M-HL drafted the manuscript and were involved directly in treating the patient. YP provided figures and formalized the manuscript. HY, T-HK, J-SU, BJ, and H-NP reviewed the drafts and made contributions to proofreading. All authors approved the submitted version.

## Conflict of Interest

The authors declare that the research was conducted in the absence of any commercial or financial relationships that could be construed as a potential conflict of interest.

## Publisher’s Note

All claims expressed in this article are solely those of the authors and do not necessarily represent those of their affiliated organizations, or those of the publisher, the editors and the reviewers. Any product that may be evaluated in this article, or claim that may be made by its manufacturer, is not guaranteed or endorsed by the publisher.

## Supplementary Material

Supplementary Figure 1 Pre-procedural cardiac computed tomography short-axis **(A)** and 2-chamber **(B)** cines reveal no evidence of aneurysm or structural defect at baseline.

Supplementary Video 1 Intracardiac echocardiography showing steam pops and termination of the ventricular tachycardia.

Supplementary Video 2 A 2-month follow-up transthoracic echocardiographic view of the delayed left ventricular pseudoaneurysm.

## Abbreviations

CT: computed tomography
ICE: intracardiac echocardiography
LV: left ventricle
VT: ventricular tachycardia.
