# Supplementary figures and images for: Case Report: Delayed Ventricular Pseudoaneurysm After Radiofrequency Ablation of Left Posteromedial Papillary Muscle Ventricular Tachycardia
Source: Front Cardiovasc Med. 2022 Jun 15;9:887190. doi: 10.3389/fcvm.2022.887190 (PMC9240706; doi:10.3389/fcvm.2022.887190)

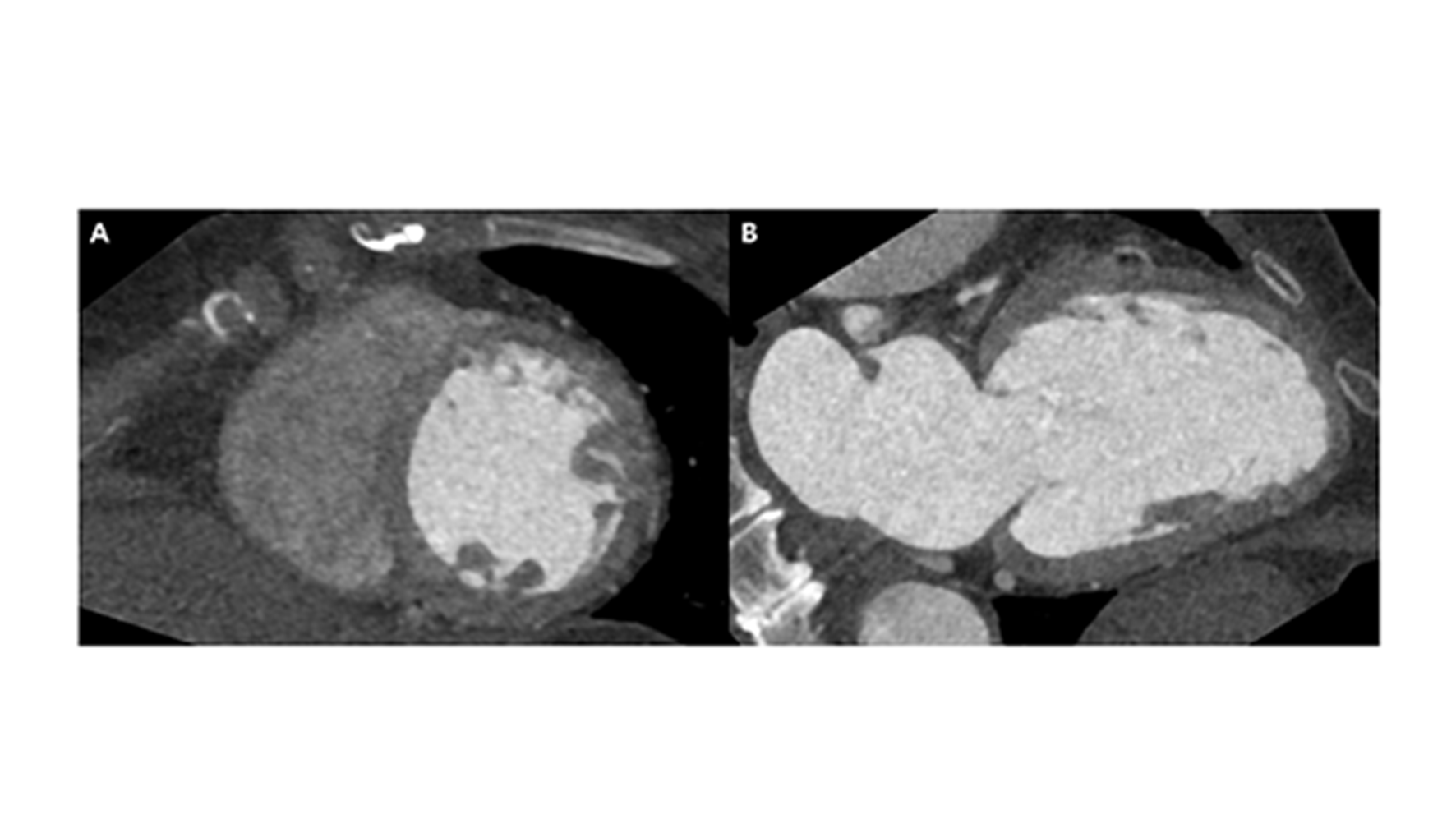

Supplement: Supplementary Figure 1 — Pre-procedural cardiac computed tomography short-axis (A) and 2-chamber (B) cines reveal no evidence of aneurysm or structural defect at baseline. [file Image_1.TIF]
